# Effectiveness of isosorbide dinitrate in cyanide poisoning as a function of the administration timing

**DOI:** 10.1186/s40360-017-0122-0

**Published:** 2017-03-14

**Authors:** Ophir Lavon, Amit Avrahami, Arik Eisenkraft

**Affiliations:** 1 Clinical Pharmacology and Toxicology Unit, Carmel Medical Center, 7 Michal St., Haifa, 3436212 Israel; 2 Rappaport Faculty of Medicine, Technion-Israel Institute of Technology, Haifa, Israel; 3 Pre Clinical Research Authority, Technion-Israel Institute of Technology, Haifa, Israel; 4 Institute for Research in Military Medicine, Faculty of Medicine, Hebrew University, Jerusalem, Israel

**Keywords:** Cyanide, Isosorbide dinitrate, Antidote, Poisoning, Animal

## Abstract

**Background:**

Better and safer antidotes against cyanide poisoning are needed. Prior study has shown a favorable effect of isosorbide dinitrate (ISDN) on the survival of cyanide-poisoned rabbits when administered as early as 1 min after poisoning. The aim of the current study was to further evaluate the efficacy of intravenous ISDN administered in clinically relevant timing for first responders.

**Methods:**

A comparative animal study using 24 rabbits in 4 randomized study groups was performed. Animals were poisoned with intravenous potassium cyanide (1 mg/kg). Animals in Group 1 served as controls and received no treatment. Groups 2–4 animals were treated intravenously with ISDN (50 μg/kg) after poisoning; one group after 3 min, another group after 5 min and the last after 7 min. Animals were observed for 30 min after poisoning. The study endpoints included survival rate, clinical status, blood pressure, pulse per minute, blood lactate and pH.

**Results:**

Five of 6 animals (83.3%) from every treatment group survived the whole observation period while all control untreated animals died. All the rabbits collapsed immediately after exposure, showing rapidly deteriorated vital signs with lactic metabolic acidosis (peak blood lactate levels of 18.1 to 19.0 mmol/L on average at 10 min post exposure). Vital signs, clinical scores, and blood gases of treated rabbits gradually improved.

**Conclusion:**

Poisoned rabbits showed improved short-term survival following the administration of ISDN up to 7 min after lethal cyanide poisoning of. We see a potential for ISDN as an antidote against cyanide poisoning.

## Background

The toxic chemical cyanide is frequently used in different industries [1, 2]. Cyanide poisoning poses an imminent threat in various scenarios, both intentional and accidental [1, 3, 4]. Currently used antidotes have several major limitations. The expensive Hydroxocobalamin exerts its effects when given intravenously, and within 10 to 15 min [5]. Another intravenously administered antidote is sodium thiosulfate, which also acts as a relatively slow antidote [6]. Nitrites have significant adverse reactions such as hypotension and profound dose-dependent methemoglobinemia. They also have relative contraindications in certain scenarios and populations [3, 6]. In a similar way, dicobalt edetate is given intravenously and has several safety issues [1, 6].

In a previous study we demonstrated that administration of intravenous isosorbide dinitrate (ISDN) within 1 min after lethal cyanide poisoning of rabbits improved their survival with full recovery of blood gases, vital signs and clinical scores [7].

ISDN, is a nitrate used pharmacologically as a vasodilator for angina (heart-related chest pain), congestive heart failure, and esophageal spasms [8].

The aim of the current work is to test whether the efficacy of intravenous ISDN depends on the timing from poisoning to therapy.

## Methods

We have conducted a clinically relevant animal study using a methodology previously reported [7]. Twenty four rabbits were studied, divided into 4 study groups. Group 1 animals were not treated after poisoning and served as controls. The animals of groups 2–4 were treated intravenously with ISDN (50 μg/kg) after poisoning; group 2 after 3 min, group 3 after 5 min and the group 4 after 7 min. These doses are weight-equivalent to human doses. The study endpoints included survival rate, clinical status (presented by a score that was developed for our previously reported study), pulse per minute, BP (blood pressure), blood lactate, and pH. The observation time was up to ½ an hour. Prior to the start of each research day the poison solution and the medication were specially prepared in a method previously reported [7]. Appropriate vascular accesses were prepared in the rabbit ear. Arterial blood probe was attached to monitor the vital signs. This monitoring system was successfully used in our previously reported study [7].

The calculated cyanide poison dose was intravenously injected. The ISDN was intravenously administered 3, 5 or 7 min after poisoning (depending on the study group). During the continuous observation, outcomes were recorded in time points set in advance; vital signs every minute, clinical score at 0, 1, 2, 3, 4, 5, 7, 10, 15, 30 min, and blood sample at 0, 2, 5,10, 15, 30 min. The clinical score included 6° from death (0) to fully alert and moving (5). Blood sampling and laboratory analysis was completed in a previously reported method [7].

Comprehensive data analysis was conducted using SPSS Statistics version 22, IBM®.

## Results

**Fig. 1:**
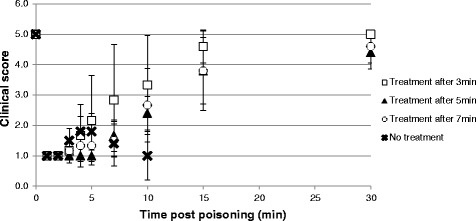
Mean clinical scores (± SD)

Five of 6 rabbits (83.3%) from every treatment group survived the whole 30 min observation period. All untreated animals died. The single animal per treatment group that did not survive died in the time range of 8 to 13 min after poisoning; the untreated control animals died in a median time to death of 8 min (range 2 to 15 min). All rabbits collapsed within 1 min of exposure, with the clinical score falling sharply from 5 to 1. All rabbits showed generalized tonic-clonic convulsions, most probably seizures, within 25 to 40 s of exposure; clinical status then deteriorated into complete unresponsiveness. The animals treated 3 min after poisoning had a trend for better clinical scores when compared to animals receiving the treatment later, from 4 min after exposure and until the end of observation. This was not statistically significant when evaluated with One-Way ANOVA test. Time vs. average clinical scores are shown in Fig. 1.

**Fig. 2:**
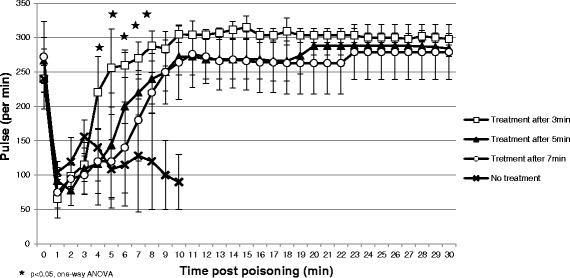
Mean pulse (beats per min ± SD)

**Fig. 3:**
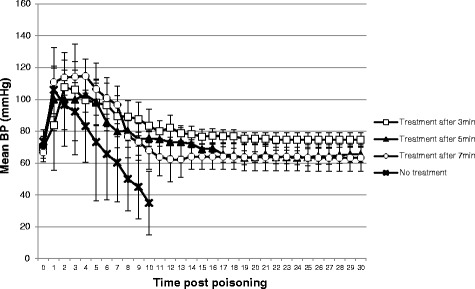
Mean blood pressure (mmHg ± SD)

We found a considerable drop in pulse post exposure from average pulse rates of 240–272 per min pre-exposure to 66–104 per min after 1 min. Mean BP increased from average values of 67–74 mmHg before poisoning to 84–111 mmHg 1 min after intoxication. Steady improvement of BP and pulse to baseline levels was found in all treated rabbits, while several variations were seen between groups. Pulse values at 4 to 8 min post intoxication were significantly higher in the 3 min group compared to the other groups (*p* < 0.05, One-Way ANOVA). Average mean BP and pulse data are presented in Figs. 2 and 3.

**Fig. 4:**
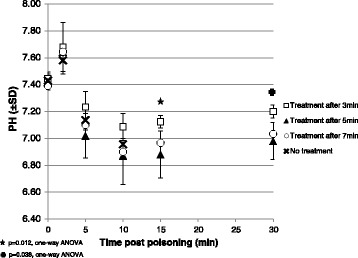
Mean blood pH (± SD)

Shortly after poisoning, blood pH increased, representing a self-limited phase of hyperventilation, and then fallen to profound acidosis. Steady improvement was observed in all treated rabbits, yet baseline pH levels were not reached until the end of the observation period. Animals treated 3 min post exposure had significantly higher and closer to normal range pH values at the 15 and 30 min after poisoning time points (*p* = 0.012 and *p* = 0.038, respectively, One-Way ANOVA). Results of blood pH are shown in Fig. 4.

**Fig. 5:**
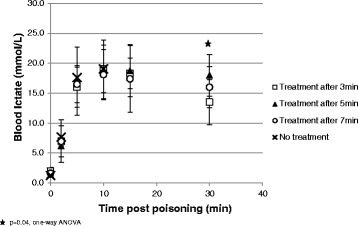
Mean blood lactate (mmol/L ± SD)

We found a gradual increase in lactate levels, peaked 10 min after poisoning in all groups. A slow return towards baseline levels was found in all treated rabbits but it did not return to baseline until the end of the observation period. Lactate levels were significantly lower and closer to normal range in the 3 min treatment group compared to the other later treated groups at 30 min post poisoning (averages 13.5 vs. 17.3 and 18 mmol/L, *p* = 0.04, One-Way ANOVA). Average lactate results are presented in Fig. 5.

## Discussion

The study results demonstrate clear effectiveness of intravenous ISDN administered up to 7 min after lethal cyanide poisoning in increasing the short-term survival of poisoned rabbits. This observation strengthens previous study results that showed survival improvement with ISDN given 1 min after cyanide poisoning in rabbits [7]. The importance and contribution of the current study is the evidence it provides for ISDN clinical effectiveness in a realistic time frame for first responders (estimated to be several minutes) and its relevance as an antidote for cyanide. ISDN effectiveness was demonstrated in clinically relevant doses (50 μg/kg), supporting its role as a potential medical countermeasure in emergency clinical settings.

The vital signs (both pulse and BP) and clinical scores of the treated rabbits improved steadily after receiving the drug while untreated animals deteriorated until death. Earlier treatment (3 min after poisoning) improved relatively faster the clinical status of the animals back to baseline. Statistically significant difference between the 3 min group and the other two treatment groups was calculated for the pulse values at 4 to 8 min after poisoning; this may be an evidence for the superiority of early treatment. Nevertheless, there were no statistically significant differences between the treatment groups in any of the other clinical endpoints including clinical scores, BP values and pulse rates beyond 8 min post poisoning. This represents meaningful and beneficial effects of ISDN administration up to 7 min post poisoning, not only on mere survival but also on clinical parameters.

The recovery of blood lactate and pH levels in the treated rabbits was less obvious than the improvement of the clinical endpoints but still evident. Baseline values were not achieved up to the end of the observation period. It seems that metabolic improvement is lagging after the clinical course. Perhaps a longer duration of observation will demonstrate full improvement of pH and lactate to normal range. Significant differences in pH (at 15 and 30 min post poisoning) and lactate (at 30 min post poisoning) levels were observed between the earlier treated animals to the other two treatment groups; early treatment is superior in improving lactic metabolic acidosis after cyanide poisoning. Yet, even later treatment of up to 7 min after poisoning resulted in the improvement of acidosis.

The presented animal model correlates well with a human poisoning. The main mechanism of cyanide poisoning in humans and other mammals include the inhibition of cytochrome oxidase C and the impairment of the aerobic metabolism in the mitochondria [1]. Cyanide toxicity occurs because this compound strongly binds to metals, inactivating metalloenzymes such as cytochrome c oxidase [2]. cyanide causes apoptotic cell death that is caspase-dependent and associated with mitochondrial membrane depolarization and cytochrome c release [9].

Lethal Cyanide poisoning exerts a distinctive clinical course [7, 10]. This was clearly demonstrated in the current study. Exposure to cyanide leads to a drastic initial elevation of BP, probably due to vasoconstriction. This sharp increase in afterload can increase the oxygen demand and the heart’s load, thus impairing cardiac output. This may lead to myocardial ischemia and bradycardia.

Another prominent clinical observation was the early development of seizures. Cyanide, through disruption of brain glutamatergic transmission, induces apnea and seizures [11]. In rats, within 30 s after exposure to cyanide, glutamate levels increase in several brain regions [12]. Inward currents produced by the NMDA receptor which can potentiate glutamatergic activity are also enhanced by cyanide [13].

Nitrates are rapidly reduced to nitric oxide (NO) in blood and in tissues, in a NO synthase-independent pathway [14, 15]. The production of NO is increased gradually as oxygen tensions falls [16, 17]. NO leads to vasodilatation, that can improve vascular bed ischemia, via the relaxation of muscle cells [18, 19]. Vasodilatation reduces the afterload (arterial pressure) and the preload (the pressure of the blood returning to the heart) [20]. NO may also increase the oxygen supply to the heart, through reduction of coronary spasm. The administration of nitrates as ISDN in acute coronary events, ameliorates myocardial ischemia by improving coronary flow and decreasing cardiac workload [21, 22]. Phenoxybenzamine and chlorpromazine — other vasodilators—can also improve survival after exposure to cyanide [23–25]. NO-induced vasodilatation seems to have a beneficial role in cyanide intoxication.

The severity of the lactic acidosis as expressed by the blood lactate and pH levels was not different between treated and untreated animals in the study while a clear difference was observed regarding the vital signs’ dynamics. It seems that the circulatory improvement of the treated animals was better predictive and correlated with their survival compared with their metabolic status. This supports the suggested mechanism of action of ISDN in cyanide exposure, improved circulation.

NO has direct antagonistic effect on cyanide in the mitochondrial electron transport chain, as it competes with it for binding to cytochrome c oxidase [26, 27]. Leavesley et al. demonstrated in an animal model that addition of exogenous NO attenuated cyanide inhibition of cytochrome c oxidase [27]. The same was observed when sodium nitrite was used as the source of NO that interacted directly with the binding of potassium cyanide with cytochrome c oxidase to reverse toxicity [28]. Also, it seems as if NO protects enzymatic systems from cyanide inactivation via a mechanism involving the formation of an enzyme-nitrosyl cyanide complex [29].

Brain NMDA-receptor hyperactivity reduction is another potential mechanism of NO protection in cyanide poisoning. NO+ ion protects from excessive stimulation of the NMDA receptor by binding to its redox regulatory site [30]. This may attenuate repeated seizures as seen in the current study after ISDN administration.

The direct and independent antidotal activity of NO in cyanide poisoning is probably a more important and faster mechanism of nitrites and nitrates as cyanide antidotes than methemoglobin formation. Several animal and human studies showed that nitrites (sodium and amyl) improved survival in cyanide poisoning without forming significant levels of methemoglobin [7, 11, 31–33]. Production rate of methemoglobin by nitrites is relatively slow in humans [34]. The reported early recovery of severe cyanide poisoned victims following nitrite treatment is too fast to be explained by methemoglobin formation [31, 35, 36].

While nitrates in therapeutic doses do not form methemoglobin, they were not considered relevant for cyanide poisoning. However, as it is shown and proven that NO antagonism is a principle mechanism in cyanide treatment, nitrates should be incorporated in the protocols implemented in cyanide exposure and poisoning particularly in the field setup or in mass casualty incidents.

Nitrates present several advantages over currently used antidotes. Nitrates are an approved, safe, and low cost treatment, easily available and widely used; particularly in cardiac patients [37–40]. There are various rapidly absorbed formulations with easy and effective application in pre-hospital setups, even by laymen [38]. Nitrates have no significant interactions with other cyanide antidotes or resuscitation medications [37, 38].

The study has several limitations. It is a limited number rabbit study with a short-term follow up period. Exposure to cyanide was achieved in the intravenous route, which is not the usual human exposure route. The investigators were not blinded to the control and treatment groups.

## Conclusion

Intravenous administration of ISDN up to 7 min after minimally lethal cyanide poisoning improved the short-term survival of poisoned rabbits. ISDN shows marked potential as an antidote for cyanide poisoning. Further research is needed to evaluate the effectiveness of nitrates in other modes of delivery, different animal models and poisoning scenarios.
